# The influence of age and gender on self-assessment of piano competencies

**DOI:** 10.3389/fpsyg.2025.1671900

**Published:** 2026-02-20

**Authors:** Chen Chen, Wen Lin

**Affiliations:** Music Department, Guangzhou Xinhua University, Guangzhou, China

**Keywords:** age differences in learning, gender differences, music education, piano playing, self-assessment of skills

## Abstract

**Introduction:**

This study examined how pianists assess their own abilities in four areas: technical proficiency, musical understanding, expressive control, and presentation skills.

**Methods:**

Participants included piano students and professional pianists, all with at least ten years of experience. Results were compared across age and gender groups. The study involved 600 participants divided into three age groups: 13- to 15-year-olds (M = 14.2, SD = 2.01), 23- to 24-year-olds (M = 23.8, SD = 2.24), and 36- to 42-year-olds (M = 40.4, SD = 5.38). Data were collected using the Bem Sex-Role Inventory and a custom-designed piano competency questionnaire. The statistical significance of differences between men and women in their self-assessment of performance skills was then tested.

**Results and discussion:**

Among music college students (mean age 23.8 years)—the only group where a significant gender difference was found—men rated their technical skills higher than women did. The assessment of other subscales revealed no statistically significant differences between male and female piano players. Overall, the data do not support the idea that gender influences how pianists evaluate their own competencies. In contrast, age differences proved significant across all scales of piano competency (*p* = 0.00). The results indicate that pianists in older age groups rate their technique, musical literacy, expressiveness, and stage skills more highly. This increase in self-assessment is directly correlated with years of practice. The present findings can be used by music educators, parents, piano enthusiasts, and professional pianists. By understanding how age and gender shape pianists' self-perception, educators can tailor teaching methodologies to align with how students perceive their own skills.

## 1 Introduction

The piano is a widely popular musical instrument (Shi, 2024), and learning to play it is crucial for people who make music (Franceschi, 2022). Practicing the piano allows for self-expression and improves cognitive abilities. Musicians must read music sheets, coordinate their hands and fingers, and maintain rhythm. The repetitive nature of piano scales and complex pieces helps to improve motor skills, and it deepens the understanding and appreciation of different musical styles, composers, and historical periods (Guo et al., 2022; Hung et al., 2021; Wu, 2020). The ability to express oneself through the harmonious combination of notes has a universal appeal, encouraging people from all over the world to learn the piano (Li, 2020).

Individuals possess varying aptitudes for piano performance. Within the context of this research, self-assessment of piano competency refers to a pianist's subjective perception of their own skills in technical execution, musical expressiveness, and music creation (Kusumaningsih and Ibrahim, 2020). This concept encompasses both professional techniques and general musical abilities. There are certain technical, musical, and expressive nuances that piano players must account for. The technical side of playing the piano encompasses manual dexterity, coordination of hand and finger movements, and the accuracy of notes and rhythm patterns; to be able to play any piece of music, pianists must master the piano technique and learn the repertoire (Snell and Stringham, 2021). Musical ability in general includes the basic items related to music reading, such as the interpretation of musical notation, dynamics, phrasing, and musical structure, as well as abilities relating to audition, like having a sense of timing and rhythm. These skills allow a pianist to convey certain emotions through music and play the melodies professionally (Cheng, 2019). The expressive ability of the piano players refers to their ability to create a unique interpretation of the music piece through patterns of articulation, touch, dynamics, and pedaling (Goebl, 2018) in a way that unveils its emotional depth and character (Kondo, 2020; Smith, 2021). The performing ability, also defined as musical presentation or communication skills, includes, but is not limited to, stage presence and audience communication. It is important that pianists develop self-confidence as musicians and know how to establish a sense of connection with an audience, hold it captive during the performance, and help them understand the message of the piece (Xie, 2019).

Age is an important yet little-understood factor in the acquisition of any skill (Button et al., 2020), and piano playing is no exception, for the existing research mostly focuses on the piano-practicing abilities of children (Li, 2021) and the older population (Reifinger Jr, 2016). The role of gender also attracts considerable attention from scholars. Historically, gender stereotypes have been influencing social expectations about musical abilities, so it is important to explore the complex interaction between gender and music skills to uncover how gender shapes the piano learning experience of individuals (Gray, 2022; Gurel, 2022). Previous research on the influence of age and gender on piano learning indicates that these factors can significantly impact achievements in music education (Herrera and Cremades, 2020). Scholars have identified that children and adults learn differently, with younger ages often associated with higher adaptability to new teaching methodologies (Liquin and Gopnik, 2022). Gender also plays a role, as studies highlight differences between males and females, emphasizing the importance of an individualized approach to music education, considering the age and gender variations among students (Gray, 2022).

Learning to play the piano is a complex task: it requires constant practice, guidance from an experienced teacher, and exposure to a diverse repertoire (Ma and Ma, 2023) and variables, such as age and gender, bear an influence on the development of piano abilities (Dempsey and Comeau, 2019). This article examines the relationship of age and gender with the self-assessment of pianistic skills, utilizing a questionnaire for data collection. The necessity of conducting this research is supported by the desire to address a critical gap in understanding the nuances of the roles of age and gender in piano playing skills. Analyzing pianists' self-evaluations provides empirical data regarding their subjective perception of ability. Such information can form the basis for novel pedagogical approaches, making the identification of factors influencing pianists' self-esteem a relevant pursuit. Valuable insights can be gleaned from this information, and it can be used to inform instruction. The study contributes to the current knowledge base in the fields of music education and developmental psychology by providing a platform for identifying age- and gender-driven patterns and trends in piano education.

### 1.1 Literature review

Age and gender categories are ascribed attributes and traits that are segmented by age, gender, and related stereotypes, and these characteristics can potentially affect the development of any musical ability (Güçlütürk and van Lier, 2019). The age categories cover three developmental domains: physical, cognitive, and socio-emotional, which change as a person grows and thus require different approaches to teaching; for instance, childhood ages can benefit from playful and interactive learning (Gorbunova and Hiner, 2019). On the other hand, adults may bring previous musical experience, discipline, and greater self-regulation into the learning process, and may not be suited to the teaching method used with children (Chaddock-Heyman et al., 2021).

Some ages constitute critical periods in musical development, in the sense that there are certain age ranges where musicians can acquire musical abilities more easily (Lamont and Hargreaves, 2021). Yet, there is still debate on whether this is the case. Age can influence the formation of piano practicing abilities due to various factors; for instance, younger piano players may have greater neuroplasticity than older pianists, which allows them to acquire more technical skills (Weyandt et al., 2020), yet they still may struggle to understand complex musical concepts and convey the emotional depth of music (Barrett et al., 2019). Experienced musicians tend to be more disciplined and musically mature than beginners, which underlies their higher self-rating of skills. Also, one's age can affect their ability to devote time and effort to practice, as well as the ability to assimilate new information and adapt to new methods (Marufjon, 2020; Shafoatovich, 2021).

Gender refers to social and cultural expectations, roles, and stereotypes associated with being a man or a woman (Trollinger, 2021). It may influence a person in choosing the musical instrument, as well as affect their musical preferences and perceived musical ability (Xu et al., 2021). In theory, all genders can excel at playing the piano in practice; therefore, gender does not determine one's skills or abilities, but even so, a certain effect still exists (Gurel, 2022).

The piano skills needed for expressive playing are complex and multifaceted. To this day, there is no unified way to evaluate the piano practicing ability, for it covers a wide range of different characteristics (Kim et al., 2021), and the evaluation process itself relies on self-reports or subjective opinions of the evaluator (Pati et al., 2018). This study divides piano practicing ability into four components: technical proficiency, musical understanding, expressive control, and presentation skills, which are not universally yet important for piano playing (Fabijanić, 2021).

The mastery of piano technique is based on building the dexterity of finger and hand movements as well as fine motor coordination. These fundamental technical skills allow pianists to play scales, arpeggios, chords, and complex passages at any speed with great precision (Ömür, 2022). The correct hand position, finger independence, and the efficient use of both hands form the foundation of the piano playing technique (Kim et al., 2021). Overall, technical skill determines musical expression, as it is vital to the precision and expressiveness of the performance (Fratila, 2021). Once automaticity with technical skills is achieved, piano players can focus on experimenting with different tonal colors and musical ideas, and on expanding their repertoire (Ömür, 2022; Sklyarov, 2020).

Musical understanding implies the knowledge of music theory, including scales, chords, harmony, rhythm, and notation (Pfordresher, 2022). Understanding the musical structure, dynamics, and phrasing allows pianists to bring out the essence of a music piece and convey its intended meaning (Imai-Matsumura and Mutou, 2021). Musical understanding is essential for pianists because it enables them to delve deeper into music, make informed choices, and effectively communicate the composer's intentions. Pianists with good musical understanding can “make sense” of music, emphasize the contrasts, maintain a coherent music flow, and bring out different musical nuances during a performance (Coppola, 2021; Cota, 2019).

The expressive ability of musicians is about interpretation: piano players make use of their personal feelings and musicality to bring music to life (Alper, 2021). Essentially, it is the ability to make informed decisions about tempo, dynamics, and articulation to convey the expressive intentions of the music. For this to be possible, piano players must understand the composer's style and the historical context behind a piece. Another vital requirement is creativity (Bonnaire and González-Moreno, 2023). Pianists with excellent expressive control can connect with music and the audience and bring originality to their performance (Tetsuya and Kei, 2020).

Playing the piano goes beyond playing the right notes and rhythms: piano players must also possess presentation skills, such as stage presence, body language, and audience communication. The expressive body movements and physical gestures are essential for engagement and a compelling experience (Suzuki and Mitchell, 2022; Tominaga et al., 2022) for they help to effectively communicate the intended message of the music piece. Piano players with remarkable presentation skills can keep calm and confident when on stage, be one with the music, and deliver musical interpretations in a way that is entertaining (Iseev, 2022).

### 1.2 Problem statement

Factors such as age and gender can potentially influence a person's ability to learn the piano. This study seeks to explore and understand how these factors affect piano learning to enhance music education. This study aims to identify the influence of age and gender on the self-assessment of four pianistic competencies: perceived technical proficiency, musical understanding, expressive control, and presentation skills. The secondary objective is to assess the statistical significance of intergroup gendered differences in competency between different age groups.

## 2 Materials and methods

This study has developed a 40-item questionnaire with four blocks dedicated to a particular piano competence. The blocks were numbered accordingly: (1) Technical Proficiency; (2) Musical Understanding; (3) Expressive Control; and (4) Presentation Skills. Each contains 10 equations. It takes 45 mins to complete. The score range was 10 to 40 per section. The questionnaire was in Chinese and used a 5-point Likert scale ranging from 1 (strongly disagree) to 5 (strongly agree). This instrument is based on self-reporting, not on objective performance metrics, and records respondents' subjective perceptions of their own pianistic competencies. Accordingly, the questionnaire captures subjective self-assessment and does not address objective levels of mastery. The choice of a self-report method is justified by the central role that self-perception of skill plays in musicians' motivation and professional trajectories. The methodological decision to employ self-reports enables the study of pianists' self-perception, which often diverges from objective performance tests (Pati et al., 2018). The study also used the Bem Sex Role Inventory, or BSRI, to identify gender differences between students (Bem, 1974). The BSRI was translated into the native language of the respondents–the Chinese language.

### 2.1 Validation and reliability of instruments

Before implementation in the main study, the questionnaire, developed by professionals in the field of piano music education, underwent pilot testing with a small group of respondents (*n* = 50). This stage allowed the identification and rectification of potential inconsistencies and shortcomings in question formulations, as well as the verification of participants' understanding and interpretation of the rating scale. After collecting and analyzing preliminary data, a factor analysis was conducted, confirming that each block of questions adequately reflects the measured competence. To assess the reliability of the instrument, Cronbach's alpha coefficients were calculated for each block of questions, with values (0.90, 0.93, 0.90, and 0.92, respectively) indicating high internal consistency and reliability of the questions within each block. Additionally, to assess gender differences, Bem's Sex Role Inventory (BSRI) was used, adapted, and translated into Chinese to fit the research context. Validation of the translated version of BSRI included checking its internal consistency, with a coefficient of 0.91, confirming the high reliability of the instrument in the context of this study. For further validation of both instruments, survey results were compared with predetermined theoretical assumptions and factor analysis indices were calculated, allowing not only confirmation of the reliability of the methods used but also ensuring their validity through comparison with theoretical frameworks in the field of piano competencies and gender studies. Self-assessed skill levels often differ from objective indicators of performance quality.

### 2.2 Participants

The study involved 600 respondents divided into three equal groups: teenage piano students from a local piano school, fifth year piano students from one of the national universities, and professional pianists with at least 10 years of piano experience. Students were recruited orally through their educational institutions and via WeChat. Professional musicians were invited to participate in the study via email and social networks. The study employed a method of stratified sampling, wherein all participants were evenly distributed across three groups to ensure a comprehensive analysis of various stages of musical development. This sample size was chosen based on statistical power analysis to ensure sufficient statistical significance and reliability in detecting differences between groups. More details can be seen in Table 1 below.

**Table 1:** Age and gender distributions of the participants.

**Group**	**Age**	**Total**	**Man**	**Woman**
A	13-15 years	200	100	100
B	23-24 years	200	100	100
C	36-42 years	200	100	100

Participants in group A ranged in age from 13 to 15 years (M = 14.2, SD = 2.01). The age of the participants in group B ranged from 23 to 24 years (M = 23.8, SD = 2.24). Participants in group C were 36 to 42 years of age (M = 40.4, SD = 5.38). The selection criteria for participants were carefully deliberated to ensure the representativeness of the sample and the potential for generalizing the results to a broader population of musicians. Primarily, participants were selected based on their musical education and experience. For adolescent students from a specialized piano school, the criterion was a minimum of 3 years of intensive piano instruction. Fifth year university students were required to be enrolled in music education programs with a focus on classical piano, while professional pianists needed to have a minimum of 10 years of practical performance and teaching experience in piano playing. An important condition for participation was the provision of confirmed informed consent, obtained directly from the participants and, for underage participants in Group A, also from their parents or legal guardians. To ensure diversity and breadth in the study, it was decided to include only participants who were Chinese and received musical education in China, allowing for a focus on the specificity of learning and practice in this cultural and educational context. Additionally, it was crucial to ensure that participants did not have serious hearing impairments or neurological disorders that could affect their musical skills or ability to participate in the study. To achieve this, a preliminary screening was conducted using questionnaires and medical records. Furthermore, participants' willingness and motivation to participate in the study were taken into account during the selection process, which was particularly important for ensuring the quality and reliability of the collected data. Participants were informed about the objectives of the research, its potential outcomes, and how the gathered information would be utilized.

### 2.3 Study design

The current study, conducted in April 2023, represents a cross-sectional analysis involving participants divided into three age groups: adolescents (13-15 years), young adults (23-24 years), and mature adults (36-42 years). Special attention was paid during participant selection to ensure equal representation of each age category. Data collection was carried out online in specially equipped computer labs. The use of an online format allowed standardization of the study conditions and the simultaneous collection of data from a large number of respondents. To assess pianistic skills, a questionnaire comprising 40 questions divided into four blocks, each aimed at studying various aspects of piano performance abilities, was developed and administered. Before the survey, all participants were informed about the filling procedure, after which they were provided with 45 mins to respond. This approach facilitated careful consideration of each answer, minimizing potential stress from time constraints and enhancing the accuracy of self-assessments. Statistical data analysis aimed to identify differences in pianistic abilities among different age and gender groups.

### 2.4 Data analysis

Pianist groups were compared in terms of technical proficiency, musical understanding, expressive control, and presentation skills. For this, the Mann-Whitney U-test for independent samples and the Kruskal-Wallis test were used. Results are presented as means, standard errors (SEM), standard deviations (SD), kurtoses, and ranges.

### 2.5 Ethical issues

The research was conducted ethically in accordance with the World Medical Association Declaration of Helsinki. The study protocol was approved by the Ethics Committee of the Guangzhou Xinhua University, protocol number–GCTBSMEC2023_48271038471_2 dated April 2023. All participants were informed about the goals and objectives of the study. Their participation was voluntary. Anonymity was guaranteed. All the required permissions from parents and legal guardians for including underaged children in the study were obtained by the authors of this research.

## 3 Results

Table 2 presents gendered scores and descriptive statistics for each piano competence in teenage pianists (13 to 15 years of age). Among male participants aged 13-15, the highest self-assessment score was recorded for Expressive Control (21.75). This was followed by scores for Musical Understanding (21.16), Technical Proficiency (19.96), and Presentation Skills (17.36). The SEM values range from 0.316 to 0.461.

**Table 2:** Distribution of self-assessed piano competencies among male and female pianists in Group A (mean age 14.2 years) with descriptive statistics.

**Gender**		**Technical proficiency**	**Musical understanding**	**Expressive control**	**Presentation skills**
Male	Mean	19.96	21.16	21.75	17.36
	SEM	0.316	0.377	0.461	0.323
	SD	3.162	3.773	4.609	3.230
	Kurtosis	−1.255	−1.313	−1.206	−1.024
	Range	10	11	14	11
Female	Mean	20.09	21.53	21.45	17.30
	SEM	0.303	0.337	0.408	0.374
	SD	3.032	3.368	4.076	3.740
	Kurtosis	−1.234	−1.113	−0.979	−1.342
	Range	10	11	14	11

SEM is the standard error of the mean; SD stands for standard deviation.

Female pianists demonstrated superior results in Musical Understanding with a score of 21.53. Expressive Control ranked next (21.45), while Technical Proficiency and Presentation Skills both received identical scores of 20.09. The gendered self-reported scores and related statistics for the group of respondents aged 23 to 24 years are presented in Table 3.

**Table 3:** Distribution of self-assessed piano competencies among male and female pianists in Group B (mean age 23.8 years) with descriptive statistics.

**Gender**		**Technical proficiency**	**Musical understanding**	**Expressive control**	**Presentation skills**
Male	Mean	23.61	26.28	25.53	24.00
	SEM	0.256	0.206	0.288	0.202
	SD	2.562	2.055	2.883	2.015
	Kurtosis	−1.147	−1.381	−1.176	−1.301
	Range	8	6	9	6
Female	Mean	24.36	26.33	25.55	24.15
	SEM	0.270	0.194	0.282	0.203
	SD	2.695	1.944	2.815	2.032
	Kurtosis	−1.200	−1.106	−1.312	−1.417
	Range	8	6	9	6

SEM is the standard error of the mean; SD stands for standard deviation.

Table 2 presents the distribution of self-assessment scores for male participants aged 23-24: Musical Understanding (26.28), Expressive Control (25.53), and Technical Proficiency (24.00). Technical proficiency scored the lowest, with a score of 23.61. The SEM ranges from 0.202 to 0.288, and the SD range is 2.015 to 2.883. In Group B, female pianists achieved their highest scores for Musical Understanding (26.33). The subsequent highest score was for Expressive Control (25.55), followed closely by Technical Proficiency (24.36) and Presentation Skills (24.15). The gendered self-reported scores and related statistics for experienced pianists (Group C) are presented in Table 4.

**Table 4:** Distribution of self-assessed piano competencies among male and female pianists in Group C (mean age 40.4 years) with descriptive statistics.

**Gender**		**Technical proficiency**	**Musical understanding**	**Expressive control**	**Presentation skills**
Male	Mean	30.00	27.79	28.65	29.06
	SEM	0.207	0.138	0.103	0.140
	SD	2.069	1.380	1.029	1.399
	Kurtosis	−1.321	−1.145	−1.039	−1.240
	Range	6	4	3	4
Female	Mean	30.00	28.12	28.51	29.25
	SEM	0.199	0.141	0.111	0.136
	SD	1.995	1.409	1.105	1.359
	Kurtosis	−1.330	−1.345	−1.325	−1.242
	Range	6	4	3	4

SEM is the standard error of the mean; SD stands for standard deviation.

The respondents in Group C are professional musicians with sufficient experience. The self-assessment of Technical Proficiency in Group C reached a peak of 30.0 points, which is the absolute maximum within the entire sample. This observation is true for both genders. The second highest score in this group is the score on Presentation skills (29.06 for male pianists and 29.25 for female pianists). Both genders scored the lowest on Expressive Control and Musical Understanding. The SEM ranges from 0.103 to 0.207 for men and from 0.111 to 0.199 for women.

Table 5 shows the gendered differences between age groups across four blocks of piano competency. A statistically significant gender difference in the self-assessment of Technical Proficiency (α = 0.05) was found in Group B, which had a mean age of 23.8 years.

**Table 5:** The statistical significance in gendered differences between age groups.

**Indicator**	**Technical proficiency**	**Musical understanding**	**Expressive control**	**Presentation skills**
**Group A (M** = **14.2 years)**				
Manna-Whitney U	4887.000	4681.500	4796.500	4949.500
Wilcoxon W	9937.000	9731.500	9846.500	9999.500
Z	−0.277	−0.781	−0.499	−0.124
Asymp. Sig. (2-Tailed)	0.781	0.435	0.618	0.901
**Group B (M** = **23.8 years)**				
Manna-Whitney U	4189.500	4951.000	4978.500	4802.000
Wilcoxon W	9239.500	10001.000	10028.500	9852.000
Z	−1.993	−0.121	−0.053	−0.490
Asymp. Sig. (2-Tailed)	**0.046**	0.904	0.958	0.624
**Group C (M** = **40.4 years)**				
Manna-Whitney U	4961.000	4343.000	4635.500	4618.000
Wilcoxon W	10011.000	9393.000	9685.500	9668.000
Z	−0.096	−1.639	−0.922	−0.954
Asymp. Sig. (2-Tailed)	0.923	0.101	0.357	0.340

Other cases are statistically insignificant. Table 6 shows the overall significance of age-based differences between groups.

**Table 6:** The statistical significance in differences between age groups.

**Indicator**	**Technical proficiency**	**Musical understanding**	**Expressive control**	**Presentation skills**
Chi-square	427.702	330.926	286.894	501.804
Asymp. Sig.	0.000	0.000	0.000	0.000

Data in Table 6 above shows significant age differences on all four scales (*p* = 0.00). The results indicate that self-assessed ratings for technique, musical literacy, expressiveness, and stage skills increase as pianists mature.

## 4 Discussion

The results reflect the respondents' subjective perception of their own piano competencies. When interpreting this finding, it is essential to consider that self-assessed skill and objective measures of proficiency often diverge. The self-assessment of musical abilities is influenced by factors such as self-efficacy, social comparisons, and prior feedback experiences (Dempsey and Comeau, 2019). As anticipated, the young pianists in Group A demonstrated the lowest overall self-assessment scores compared to the older groups. An unexpected finding was that both male and female participants in this youngest group scored lowest not in Technical Proficiency, but in Presentation Skills. According to some researchers, age has a certain effect on the technical level and psychological quality of piano players, such that older pianists have better technical skills and stronger psychological endurance, while younger players have the opposite effect (Zhang, 2021). These findings coincide with the present study.

Regarding the older piano students, Group B exhibited the lowest scores on the Presentation Skills and Technical Proficiency subscales, which suggests that teens and young adults have similar issues in relation to piano competency. Hence, university education should pay more attention to these aspects of training. Exploring how to best use Internet apps in piano training, some researchers found that any technology used for this purpose must be age- and skill-appropriate to increase the intrinsic motivation of piano learners, which also indicates the influence of age in the context of piano learning (Bobbe et al., 2021). This finding reinforces the assumption that age affects the outcome of piano training.

Group C (professional pianists) received the highest scores, as expected, and their technical level and presentation skills scored the highest in relation to other piano competencies. Pianists who are older and have been learning the piano for a longer time have a more stable performance and better mental adjustment ability (Zhang, 2021). The field performance of control subjects trained in the same environment was similar compared with older subjects who had been learning to play the piano for a long time often performing better, which suggests that the age of players has a certain effect on the adjustment of their performance (Zhang, 2021). Another paper describes that for highly experienced musicians, the mechanisms used to generate creative ideas were largely automatic and unconscious, and they came from the left posterior part of the brain (Weir, 2022). By contrast, less experienced pianists rely on more analytical, deliberative brain processes in the right frontal region to develop creative melodies.

The influence of gender is also interesting. Some studies investigated the piano practicing habits of male and female vocal students by an unrelated sample test (Gurel, 2022). A significant difference was found in the posture and technique dimensions. Specifically, female students had significantly higher levels of posture and technical skills compared to their male peers (Gurel, 2022). These findings align with the significant difference between male and female piano players in Group B (M = 23.8 years) regarding the Technical proficiency score that was found in the present study. Another study found that interactive piano learning can be effective if the following criteria are met: the training course helps to develop the communication skills of piano learners; pianists are placed in a comfortable environment; teamwork is encouraged; modern multimedia technologies are in place; and highly qualified teachers are involved in the training process (Min, 2022). With that in mind, future research can investigate how the current technology can improve piano performance.

## 5 Conclusions

In group A (M = 14.2 years), male piano players scored the highest on Expressive Control (21.75), and female piano players scored the highest on Musical Understanding (21.53); both scored the lowest on Presentation Skills (17.36 and 17.30 points, respectively). In group B (M = 23.8 years), male and female piano players both scored the highest on Musical Understanding (26.28 and 26.33, respectively). At the same time, they differed regarding the lowest score they got on the piano competency survey. The male piano players scored the lowest score on Technical Proficiency (23.61), and the female piano players scored the lowest on Presentation Skills (24.15). In group C (M = 40.4 years), male and female piano players both scored the highest on Technical Proficiency (30.0) and the lowest on Musical Understanding (27.79 and 28.12, respectively). The intergroup analysis revealed that a significant difference between male and female piano players in different age groups was only present in group B (M = 23.8 years); it exists in relation to the Technical Proficiency score. No other significant gender-based differences were identified, indicating no consistent relationship between gender and the self-assessment of pianistic competencies. Another finding made in this study is the presence of significant age differences in all four piano competencies (*p* = 0.00). The results confirm that self-assessment of piano competencies (technical proficiency, musical understanding, expressive control, and presentation abilities) increases with age and experience. By casting light on the effects of age and gender variables on piano training, this study provides pianists, educators, and researchers with valuable insight into which aspects of piano performance should be improved in each age group. The age-related growth in self-assessed competence across all domains underscores the value of systematic practice and continuous learning. Educators should design instructional strategies that account for the age-specific characteristics of learners. Minimal gender differences, particularly noticeable only within one age group regarding technical skills, highlight the necessity of an inclusive approach to music education. The current study not only enriches academic understanding of the development of musical skills but also underscores the importance of adapting piano instruction to meet the diverse needs of students at different stages of their musical journey.

### 5.1 Practical implications and significance

The study's conclusions regarding the influence of age and gender on the self-assessment of pianistic competencies hold practical significance for music educators. Piano teachers can use the findings as a foundation for developing age- and gender-aware teaching strategies, with a focus on developing less established competencies. Acknowledging the influence of age on the self-assessment of pianistic skills, and the influence of gender on the self-assessment of technical mastery, paves the way for personalized recommendations. Such recommendations can help pianists maximize their potential for growth and achievement. The study's attention to the gender factor contributes to the discourse on gender equality in the music industry. The scientific novelty of this investigation lies in its focus on how age and gender influence the self-assessment of pianistic competencies. The findings may serve as a platform for further research in related fields and help achieve a deeper understanding of the multifaceted aspects that shape piano competency.

### 5.2 Limitations

A central methodological limitation is the reliance on self-reports for assessing pianistic competencies. This approach captures subjective skill perception rather than objective performance mastery. The use of self-reporting means the results reflect perceived competencies, not those measured objectively. A promising direction for future research would be to incorporate objective assessment methods, such as expert analysis of performance recordings, standardized tests of technical skills, and computer-based performance analysis. Future research may focus on identifying other factors, such as musical experience, practice habits, learning approaches, and individual learning styles, which contribute to the formation of piano practicing ability. Finally, the group of professional musicians had a larger age gap compared with the other two groups, which also seems to be the limiting factor. Additionally, no cross-cultural adaptation was conducted for the BSRI, which constitutes another limitation. The employed cross-sectional design of the study restricts the ability to establish causal relationships but allows for the identification of important trends and associations that may serve as a basis for future longitudinal research.

## Data Availability

The original contributions presented in the study are included in the article/supplementary material, further inquiries can be directed to the corresponding author.
